# Loeys-Dietz Syndrome Complicated by Right Coronary Artery Pseudoaneurysm

**DOI:** 10.1155/2018/8014820

**Published:** 2018-12-23

**Authors:** Yasir Jawaid, Obadah Aqtash, Kanaan Mansoor, Aman N. Ajmeri, Frank Fofie, Ahmed Amro, Larry Dial

**Affiliations:** MU Internal Medicine Residency Program, Marshall University, 1249 15th Street, Huntington, WV 25701, USA

## Abstract

Loeys-Dietz syndrome is a rare autosomal dominant connective tissue disorder notable for rapidly progressive vascular aneurysmal disease and craniofacial defects. Patients are at an increased risk for aneurysm rupture and dissection at younger ages compared to other aneurysmal syndromes. Early surgical intervention is important for prevention of ruptures and/or dissection. The coronary arterial tree is mostly involved as a result of postoperative complications of an aortic root repair. This fact has been sparsely reported. We report a unique case of LDS2 presenting with chest pain that was later diagnosed as a pseudoaneurysm as a result of a right coronary artery graft dehiscence.

## 1. Introduction

Loeys-Dietz syndrome (LDS) is a rare autosomal dominant syndrome which is caused by a mutation of genes encoding for transforming growth factor-beta signaling pathway *TGFBR1*, *TGFBR2, SMAD3*, and *TGFB2*. LDS is primarily a connective tissue disorder distinguished by cardiovascular, craniofacial, and skeletal defects. Hallmark findings of LDS are early and aggressive aortic and arterial aneurysms and dissection [1]. We present a case of a patient with Loeys-Dietz syndrome who underwent an aortic root replacement, which was complicated by coronary artery graft dehiscence leading to a right coronary artery pseudoaneurysm. Previous cases have described postoperative coronary artery aneurysms. We believe that a postoperative graft dehiscence presenting as a coronary artery pseudoaneurysm is a rare complication.

## 2. Case Presentation

A 60-year-old male, with a pre-existing diagnosis of Loeys-Dietz type 2, presented to the emergency room with left-sided chest pain for three weeks. He had a sudden onset of left-sided chest pain around 3 weeks ago, which was described as stabbing in nature and was 6/10 in intensity. It radiated to the back occasionally, was exaggerated by lying down, and did not increase upon exertion.

He was diagnosed with Loeys-Dietz syndrome in 2005. Genetic testing revealed a TGFBR2 mutation specifically R460H. In 2006, he underwent a prophylactic aortic root replacement for an aortic root aneurysm measuring 4.6 cm with a porcine bioprosthesis. On a routine two-year follow-up, a CT showed a left coronary artery pseudoaneurysm secondary to a left coronary artery dehiscence. He underwent another aortic root replacement and single coronary bypass with an aortosaphenous vein graft. Intraoperative findings revealed a left coronary artery sinus that had detached from the aortic graft, with increased the friable aortic tissue preventing a patch repair, necessitating an aortic replacement. In addition, other aneurysms included a right popliteal aneurysm measuring 4.4 cm, AAA measuring 3.5 cm, left internal carotid artery aneurysm measuring 9 mm, left subclavian artery aneurysm measuring 3.6 cm, and right ICA aneurysm measuring 6 mm.

**Figure 1:**
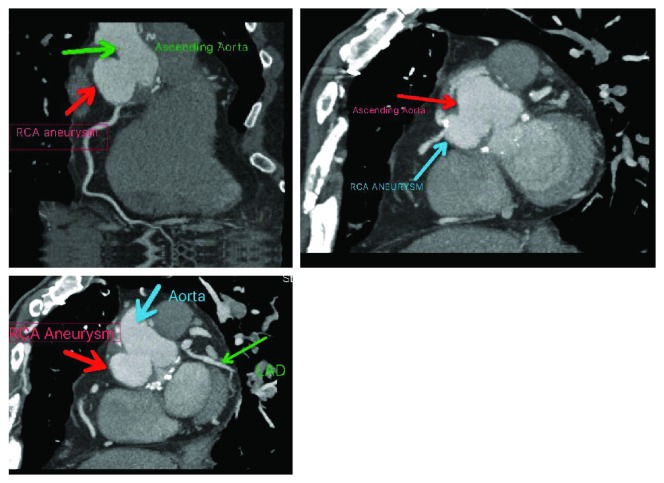
CT scan showing the right coronary artery pseudoaneurysm measuring 2.5 × 4 × 4 cm arising off the right lateral aspect of the aorta. Also shown is the sinus of Valsalva aneurysm measuring 2.4 × 3.8 × 4.0 cm in size (marked “Ascending aorta” and “Aorta”).

**Figure 2:**
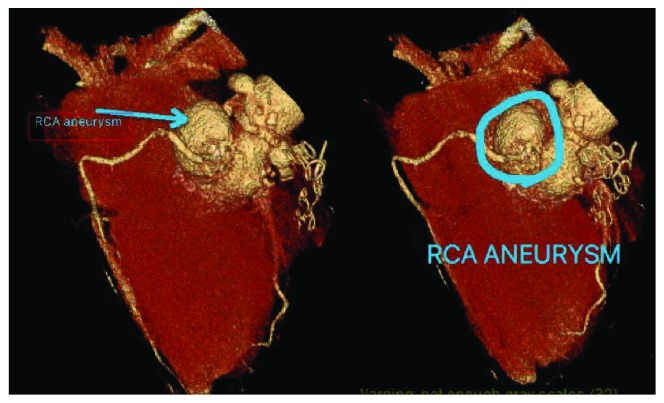
Three-dimensional reconstruction of RCA pseudoaneurysm measuring 2.5 × 4 × 4 cm arising off the right lateral aspect of the aorta.

On arrival to our emergency room, his vital signs were stable. Physical exam and electrocardiogram were unremarkable, serial cardiac enzymes were negative, and additional lab work-up was negative. Chest X-ray was obtained and was negative for any acute cardio pulmonary abnormalities. Keeping in mind his medical and surgical history, a cardiac CT scan with IV contrast was ordered which (Figure 1) revealed an aneurysm of the left subclavian artery, a sinus of Valsalva aneurysm arising from the right lateral aspect of the aorta measuring 2.4 × 3.8 × 4.0 cm and the right coronary artery arising from this aneurysm. A three-dimensional reconstruction of this pathology is shown in Figure 2. This was compared to a CT scan done approximately 2 years ago which was within normal limits, showing an aortic root replacement with stable postoperative changes. He was transferred to a specialized facility for surgical repair of the aneurysm.

He underwent a redo sternotomy where the superior vena cava was cannulated, a vent was placed in the right superior pulmonary vein, the aorta was clamped, and the heart was arrested. A pseudoaneurysm was identified and recognized to be caused by dehiscence of the right coronary artery from a large defect in the old Freestyle graft. Patch and reimplantation of the right coronary artery were considered, but this was unsuccessful in prior operations. Replacement of the old prosthesis was decided upon.

The old Freestyle was excised, and the old saphenous vein interposition graft to the left coronary was detached from it. The annulus was then rimmed with mattress sutures which were passed through the base of a Valsalva graft then a magna pericardial valve. The valve and graft were lowered, and the sutures tied in and cut. The old vein graft to the left coronary could not be directly implanted, so a segment of autologous saphenous vein was interposed. The distal end of the Valsalva graft was grafted to the ascending aorta, and the right coronary artery was anastomosed to the Valsalva.

Though the heart was resuscitated, there was significant bleeding from the undersurface of the right coronary, which was not repairable. The heart had to be rearrested, and the right coronary anastomosis was taken down. The ostium of the right coronary artery had become quite macerated, so it was oversewn, opened longitudinally, and another segment of autologous saphenous vein was grafted onto it. The proximal end of that vein graft was joined to the Valsalva graft.

The aortic clamp was released again and the heart resuscitated. The patient was warmed and weaned from bypass. Hemostasis was achieved. The heart initially seemed to be functioning normally, but when LV function worsened, it introduced the possibility that the interposition vein graft to the left coronary graft was stenotic. Accordingly, the interposition graft was revised which successfully addressed the stenosis. The heart was resuscitated again, the patient was weaned from bypass uneventfully and hemostasis was achieved. The postoperative course was unremarkable. The patient received a predischarge CT scan which showed no residual pseudoaneurysm. Coronary reimplantation grafts to the RCA and left main were patent. Additionally, the left main graft had approximately 50% stenosis due to mild kinking. The patient was ultimately discharged in a stable condition.

## 3. Discussion

Loeys-Dietz syndrome is an autosomal dominant connective tissue disorder characterized by aortic aneurysm and arterial tortuosity, hypertelorism, and bifid uvula/cleft palate. Aneurysmal disease is not limited to the aortic root and has been reported in other parts of the arterial vasculature such as vertebral, cerebral, carotid, and branches of the thoracic and abdominal aorta [1]. TGF-beta superfamily is composed of multipotential secreting cytokines which mediate normal cell growth and development, including differentiation, proliferation, motility, organization, and death. Perturbation of TGF-beta signaling is associated with vascular malformation in LDS and is manifested histologically as elastin disarray, loss of elastic fiber architecture, and increased collagen expression in the arterial wall [2].

A hallmark finding of LDS is a rapidly progressive aortic aneurysmal disease; this necessitates close monitoring [1]. Dissections of the aorta have been reported in patients as young as 3 months [3]. Surgical therapy is recommended and is dependent on the dimensions of the aorta, rate of progression, valve function, severity of noncardiac features, genotype information, and family history [4]. Dissections have been reported at aortic dimensions of 3.9-4.0 cm. As a result, in adults, surgery is recommended when the aortic root dimension is equal to or more than 4 cm or when there is a growth progression of more than 0.5 cm per year. Surgical intervention of choice is a valve-sparing surgery, as this has a low rate of complications reported and this avoids the need of anticoagulation [1].

The coronary arteries can be affected by the disease process itself or occur as a postoperative complication following an aortic root replacement. Fattori et al. reported a case of a young woman with coronary dissection extending from the left main to the proximal LAD and left circumflex artery [5]. Williams et al. reported a case of post aortic root and valve replacement (Bentall procedure) proximal RCA aneurysm [6]. Carrel et al. reported a left main coronary aneurysm following multiple aortic root replacements [7].

Patients with LDS may need multiple aortic root interventions many years following an aortic root replacement necessitating long-term surveillance. A retrospective study by Patel et al. discovered that following aortic root replacement, the freedom from reoperation at 5 years is 92% and the freedom from reoperation at 10 years is significantly lower at 54% for LDS patients [8]. This data falls in line with our patient as he required multiple aortic root replacements.

Coronary artery dehiscence is a rare complication of aortic procedures. In our patient, it may have been multifactorial. It is most commonly caused by infection of the aortocoronary vein graft suture lines, but it also occurs more frequently in patients with connective tissue disorders or in genetically defined aortopathies such as LDS. Other reasons for coronary graft dehiscence relate to more technical aspects of the Bentall procedure like increased bleeding or difficulty achieving hemostasis that may limit the reestablishment of coronary flow [9]. Pseudoaneurysm formation after aortic root replacement may also be an inherent risk in patients with LDS. A retrospective study by Liu et al. found that 4 of 31 patients that developed postoperative pseudoaneurysms requiring reoperation. They concluded that pseudoaneurysms following aortic root replacement can occur in patients with severe subtypes of LDS as all of their patients had a severe subtype with a TGFBR2 mutation [10]. This was also the case with our patient.

Our case is unique because, to the best of our knowledge, this will be the second case of an RCA aneurysm and a first report of an RCA pseudoaneurysm due to coronary artery graft dehiscence. We believe that the natural course of the disease may be responsible for this complication. Multi-institutional studies of postoperative complications following aortic root replacement in patients with Loeys-Dietz syndrome will be required to determine the incidence of coronary artery involvement.

## 4. Conclusion

Loeys-Dietz syndrome is very rare and characterized by aggressive thoracic aortic aneurysmal pathology. Surgical repair with an aortic replacement is strongly recommended but can be complicated with coronary artery involvement. Herein, we report an unusual case of coronary artery graft dehiscence leading to a right coronary artery pseudoaneurysm which has previously been sparsely reported in literature. The natural course of the disease may be responsible for this complication.
